# Effects of *Clonostachys rosea* f. *catenula* Inoculum on the Composting of Cabbage Wastes and the Endophytic Activities of the Composted Material on Tomatoes and Red Spider Mite Infestation

**DOI:** 10.3390/microorganisms9061184

**Published:** 2021-05-31

**Authors:** Nomfusi Ntsobi, Morris Fanadzo, Marilize Le Roes-Hill, Felix Nchu

**Affiliations:** 1 Department of Agriculture, Wellington Campus, Cape Peninsula University of Technology, Jan Van Riebeeck Street, Private Bag X8, Wellington 7654, South Africa; nomfusi9304ntsobi@gmail.com (N.N.); fanadzom@cput.ac.za (M.F.); 2 Department of Horticultural Sciences, Bellville Campus, Cape Peninsula University of Technology, Symphony Way, P.O. Box 1906, Bellville 7535, South Africa; 3 Applied Microbial and Health Biotechnology Institute, Bellville Campus, Cape Peninsula University of Technology, Symphony Way, P.O. Box 1906, Bellville 7535, South Africa; leroesm@cput.ac.za

**Keywords:** composting, organic vegetable wastes, inoculation, *Clonostachys rosea* f. *catenula*, tissue nutrient content, toxicity

## Abstract

Globally, fungal inocula are being explored as agents for the optimization of composting processes. This research primarily evaluates the effects of inoculating organic vegetable heaps with the entomopathogenic fungus *Clonostachys rosea* f. *catenula* (Hypocreales) on the biophysicochemical properties of the end-product of composting. Six heaps of fresh cabbage (*Brassica oleracea* var. *capitata*) waste were inoculated with *C. rosea* f. *catenula* conidia and another six were not exposed to the fungus. The composted materials from the fungus- and control-treated heaps were subsequently used as a medium to cultivate tomatoes (*Solanum lycopersicum*). The biophysicochemical characteristics of the composted materials were also assessed after composting. In addition, the protective effect of the fungal inoculum against red spider mite (*Tetranychus urticae*) infestations in the tomatoes was evaluated through the determination of conidial colonization of the plant tissue and the number of plants infested by the insect. Furthermore, phytotoxicity tests were carried out post experiment. There were few significant variations (*p* < 0.05) in heap temperature or moisture level between treatments based on the weekly data. We found no significant differences in the levels of compost macronutrient and micronutrient constituents. Remarkably, the composted materials, when incorporated into a growth medium from fungus-treated heaps, induced a 100% endophytic tissue colonization in cultivated tomato plants. While fewer red spider mite infestations were observed in tomato plants grown in composted materials from fungus-treated heaps, the difference was not significant (χ^2^ = 0.96 and *p* = 0.32). The fungal treatment yielded composted materials that significantly (*p* < 0.05) enhanced tomato seed germination, and based on the phytotoxicity test, the composted samples from the heaps exposed to the *C. rosea* f. *catenula* inoculum were not toxic to tomato seeds and seedlings. In conclusion, this study showed that *C. rosea* f. *catenula* improved the quality of composted materials in terms of fungal endophytism and seed germination.

## 1. Introduction

Numerous drawbacks, including farming practices, transportation, storage of perishable food crops, inefficient disposal, and pollution [1], are associated with the poor management of organic wastes. It is, therefore, not surprising that it has led to an increased exploration into alternative solutions to the management and beneficiation of organic wastes. Compost is an eco-friendly natural material obtained when organic plant residues are reduced to organic fertilizer and soil conditioners through a biological process known as composting [2,3]. Since fungi and bacteria are integral components of composting, it is possible to optimize the quality, biophysicochemical constituents, and yield of the end-product of composting through manipulations of fungal content in composting materials.

Reports over the years on the potential enhancement effects of entomopathogenic microbes on composting processes, though few, have opened new opportunities for managing solid organic wastes through the use of fungal inocula. Fungal species are present during the mesophilic and thermophilic stages of composting [4]. Dead organic materials serve as their host and provide energy to the fungi responsible for the breakdown of organic matter [5,6], and in the process, the fungal saprophytes enhance the biodegradation of the recalcitrant lignocellulose of plant-based organic matter [7]. Hence, certain saprophytic fungi play a vital role in the decomposition and conversion process during composting [5,8,9,10]. Interestingly, entomopathogenic fungi have also been reported to produce lignocellulolytic enzymes, such as endoproteases, aminopeptidases, esterases, and chitinases [11], all of which play crucial roles during composting [12,13]. It is therefore not surprising that entomopathogenic fungal species have been grouped among microorganisms that biodegrade or mineralize organic materials [6].

A literature survey showed that there is very little information available on fungal inoculants and their ability to optimize the biodegradation of solid raw materials to humus. For example, four mesophilic fungi, *Aspergillus niger, Aspergillus* sp. (R.), *Thrichoderma viride,* and *Penicillium* sp., that were inoculated into compost materials consisting of jowar (*Sorghum bicolor*) stalk, wheat (*Triticum aestivum*) straw (5:3), and jamun (*Syzygium cumini*) leaves, reduced the composting duration by one month and improved the quality of the composted end-product [14]. Three bacterial isolates have also been reported to improve the chemical characteristic of end-products obtained from composting [15]. Inspiring findings, such as the aforementioned, have motivated researchers to further investigate the mechanisms through which saprophytic fungi influence the decomposition of raw organic wastes, as well as the quality of the end-products of composting. Evidently, this knowledge will improve our understanding and utilization of the fungus–plant residue relationship for sustainable waste management. Some fungal entomopathogens have been reported to enhance plant growth and plant resistance to phytopathogens and herbivores [16]. Moreover, these fungi can persist in soils and growth media, while offering protection to plants and improving plant nutrition [17,18]. They also produce hormones, signaling factors [19], and nutrients [20] that can enhance plant growth.

Solid organic vegetable waste and spoilage is a serious challenge faced by many developing countries [15]. These types of residues are recalcitrant and difficult to decompose. The addition of conidia of entomopathogenic fungal endophytes to raw composting materials could potentially enhance the composting process, resulting in improved biophysicochemical characteristics, such as a high nutrient content and compost maturity. These fungi can also contribute to the production of high quality compost materials that can improve plant growth and health, and protect plants against arthropod pests; thus, contributing to the sustainable management and utilization of organic wastes. One well-known entomopathogenic fungal endophyte is *Clonostachys rosea*, a soil-borne saprophyte that belongs to the phylum Ascomycota and order Hypocreales. *Clonostachys rosea* has some interesting traits that can affect the biophysicochemical properties of composts, as well as the composting of organic wastes. For example, the fungus can biotransform bioactive compounds, breakdown starch and plastics, and produce extracellular enzymes, secondary metabolites, and hydrocarbon [21,22]. *Clonostachys rosea* f. *catenula* is a potentially ligninolytic fungus [23] and a versatile antagonist against fungal pathogens of plants [24]. The objective of this study was to evaluate the effect of *C. rosea* f. *catenula* inoculation on the biophysicochemical properties of compost, endophytic activity, phytotoxicity of composted materials, and the protective effect of the composted material containing the fungus against the insect infestation of potted tomatoes (*Solanum lycopersicum* L.) by *Tetranychus urticae* (Acari).

## 2. Materials and Methods

### 2.1. Collection of Solid Waste Materials and Preparation of Compost Heaps

Disposed fresh cabbage (*Brassica oleracea* var. *capitate*) wastes were collected from two farms (Groente Verpakkers and Schultz Vars Produkte Mark) situated in the Philippi horticultural area, Cape Town, South Africa. The collection of waste was done in the mornings (between 08:00 and 12:00), and sanitary hand gloves were used to avoid contamination. The collected wastes were placed in black polythene bags. The wastes were chopped (approx. size 2–4 cm^2^), and then thoroughly mixed to form a single big heap. Subsequently, the big heap was sub-divided into 12 equal heaps of 1 m diameter and 50 cm height. 

### 2.2. Fungal Isolate

An indigenous *C. rosea* f. *catenula* strain (SM4A), which was originally isolated from a soil sample collected from a vineyard in the Cape Winelands region [25], was used for this study. Cultures of the fungus are being maintained at Cape Peninsula University of Technology in Bellville, South Africa. Pure sub-cultures of the fungus were cultivated in half-strength Potato Dextrose Agar (PDA) containing 0.02 g/L of ampicillin (Sigma-Aldrich) and 0.04 g/L of streptomycin (Sigma-Aldrich (Pty) Ltd., Johannesburg, South Africa) in 9 and 14 cm diameter Petri dishes and incubated in the laboratory at 25 °C for 12: 12 h L:D. Four-week-old (28 days) *C. rosea* f. *catenula* conidia obtained from the PDA plates were used for inoculation of the composting materials. Mature conidia were harvested by gently scraping them off the surface of an agar plate with a spatula and transferring them onto a sterile plastic container. The conidia were suspended in 2 L glass bottles containing sterile 0.01% (*v*/*v*) Tween 80 in distilled water. The bottles were capped and mixed by agitating for five minutes by shaking and using a magnetic stirrer (at 20 °C and 300 rpm for 30 min) to form a homogenous conidial suspension. The conidial suspension was enumerated using a haemocytometer (Neubauer, Merck, South Africa) and observed with a light microscope at 40× magnification. In order to obtain the desired concentration (1 × 10^4^ conidia mL^−1^), the volume of 0.01% (*v*/*v*) Tween 80 was increased or conidia was added to the glass bottle. A conidial germination test to determine conidial viability was carried out, according to the method described by Inglis et al. [26], and a high spore germination of more than 90% was obtained.

### 2.3. Experimental Setup and Treatments

A single factor experimental design was used to test the main effect of one factor (fungus inoculum of *C. rosea* f. *catenula*) with one conidial concentration on composting materials consisting of solid raw organic cabbage wastes. This experiment was conducted in open-air conditions at the Department of Horticultural Sciences, Cape Peninsula University of Technology (Bellville Campus), South Africa. The compost heaps consisting of chopped cabbage wastes were placed two meters apart on a flat concrete surface with a black plastic lining. Six compost heaps were inoculated with a 1000 mL suspension of *C. rosea* f. *catenula* at a concentration of 1 × 10^4^ conidia mL^−1^. Each of the six control heaps received 1000 mL of sterile distilled water containing 0.01% (*v*/*v*) Tween 80 (without the fungal conidia). All the heaps were thoroughly mixed and physically turned twice a week using clean and sterile spades to increase aeration and oxygen to the microorganisms. The spades used were dipped into 2% (*v*/*v*) sodium hypochlorite solution, and then rinsed with sterile water before reuse. Each heap was randomly allocated to test and control. The experiment ran for three months. The compost heaps where uncovered and exposed to natural environmental conditions throughout the composting process. Water was equally supplied to the compost heaps using a watering can. All the heaps were watered once every two weeks and watering was stopped from nine to 12 weeks, and each of the composting mixtures received 1000 mL, with a total of 9 L of sterile water added in total. Throughout the experiment, heap temperature was monitored weekly and twice each day in the mornings and afternoons using a 1 m long thermometer probe (Major Tech). A 2-in-1 soil pH-Moisture Meter (Major Tech (Cape Agricultural Products) Cape Town, South Africa) with a humidity reference scale of 0–10 (equivalent to 0–100% humidity) was used to monitor pH and moisture content. The humidity reference scale was further classified as follows: 0–3 (low humidity), 4–7 (moderate humidity), and 8–10 (high humidity). All heap measurements were taken in the center, avoiding the side walls as these could be influenced by wind and direct sunlight. The ambient environmental conditions, such as temperature and humidity data, were recorded. The average daily data was subsequently pooled to obtain weekly means, which were eventually analyzed statistically and presented as results. 

### 2.4. Heap Color Assessment 

The color of the compost was observed and classified using the Munsell Book of color [27] as a reference at the end of the experiment. The color of compost heaps is an important physical characteristic that can be used to assess the rate of composting and maturity of the compost. 

### 2.5. Chemical Analyses of Composted Materials and Plant Tissue Nutrient Content

Chemical analyses of the composted materials were carried out by Bemlab (Pty) Ltd. Somerset West, Cape Town, South Africa. The micronutrients (B, Fe, Zn, Cu, Mn) and macronutrient (N, P, K, Ca, Mg, and C) contents were determined at the end of the trial using the methods described by Campbell and Plank [28], Miller [29], Walkley [30], and the Non-affiliated Soil Analyses Work Committee [31], as described in Mtikhulu et al. [32]. 

Tomato leaf samples were analyzed for tissue micro- and macro-nutrients at Bemlab (Somerset, Cape Town, South Africa). Leaves were washed with a Teepol solution, rinsed with de-ionized water, and dried at 70 °C overnight in an oven. The dried leaves were then milled and ashed at 480 ˚C, and shaken up in a 50:50 HCl (50%) solution for extraction through filter paper [28,29]. The K, P, C, Ca, Mg, Na, Mn, Fe, Cu, Zn, and B contents of the extracts were measured using an ICP-AES analysis method [24]. The total nitrogen (N) content of the leaves was determined through combustion in a Leco N-analyzer (Leco Corporation, St Joseph, MI, USA). 

### 2.6. End-Product Toxicity Assessments: Germination and Seedling Tests

Germination and seedling toxicity tests were carried out to assess composting end-product toxicity on tomatoes. Composted materials obtained from the control and test treatments following three months of composting were mixed with coarse river sand obtained from Stanler Farms Nursery Pty. Ltd., Cape Town, as follows: 25% fungus treated compost end-product and 75% (by volume) course river sand as test sets, and 25% untreated compost end-product and 75% (by volume) course river sand as control sets. The samples were each placed in 15 cm plastic potting containers. Sixty oxheart heirloom tomato seeds were sown in separate pots for the germination test. For the seedling toxicity test, sixty oxheart heirloom tomato seedlings were also transplanted into separate pots. Composted samples were obtained from three randomly selected heaps belonging to test and control treatments. The samples were watered once each day and maintained for eight weeks in the greenhouse under the following conditions: an average day temperature of 25 ± 5 °C and relative humidity (RH) of 65 ± 5%. 

### 2.7. Evaluating Endophytic Activity and Red Spider Mite Infestations on Tomato Plants Following Treatment of Raw Compost Materials with C. rosea f. catenula

Twenty potted plants were randomly allocated to a substrate mix containing composted materials from heaps that were pre-treated with *C. rosea* f. *catenula* or composted materials from heaps that were not pre-treated with the fungus. The soil mix consisted of 25% composting end-product and 75% coarse river sand by volume (25:75). The plants were irrigated with 250 mL of sterile distilled water by drenching each potted plant and were maintained in a greenhouse that had residual red spider mite (*Tetranychus urticae*) infestations, which were allowed to infest the plant naturally. The experiment was conducted in an environmentally controlled greenhouse (an average day temperature of 25 ± 5 °C and relative humidity (RH) of 65 ± 5%) over two months.

Red spider mite infestation levels on tomatoes were assessed in the environmentally controlled greenhouse by counting the number of plants that were infested on control and fungal-exposed treatments at the end of the experiment (two months post-treatment).

### 2.8. Re-Isolation of C. rosea f. catenula

Endophytic colonization of *C. rosea* f. *catenula* of the tomato plant leaves was assessed at 21 days by re-isolation. One leaf was carefully excised from each plant and transferred to a laminar flow cabinet in the laboratory. From each leaf, rectangular leaf sections of 1–2 mm^2^ were cut. These sections were individually surface-sterilized with 1% (*v*/*v*) sodium hypochlorite for 1 min, followed by 1 min in 70% (*v*/*v*) ethanol and rinsed twice in sterile distilled water, and then transferred onto the selective medium (half-strength PDA at 19.5 g/L containing 0.02 g/L ampicillin (Sigma-Aldrich), and streptomycin 0.04 g/L (Sigma-Aldrich, Johannesburg, South Africa)), and incubated at 25 °C. The leaf sections were visually examined on a daily basis for any presence of fungal growth: *C. rosea* f. *catenula* produces white dense mycelia that become creamy at the edge. Growth from the tissues of sterilized leaf sections was used as evidence of endophytic fungal growth. A total of 18 (9 control and 9 treatment) plants were randomly sampled and 54 leaf sections were plated equating to three leaf sections per plant. The presence of *C. rosea* f. *catenula* in at least one of the leaf sections was considered as an indication of successful plant colonization. The data was expressed in percentage colonization (number of plant replicates colonized ÷ number of plant replicates excised) × 100. 

### 2.9. Statistical Analyses

The experimental data (heap temperature, pH, moisture, compost nutrient contents, number of seeds germinated, number of live seedlings and endophytic test) were analyzed using one-way analysis of variance (ANOVA) for comparison of the control and treatment groups, while the Tukey HSD test was used to separate means at a level of significance, *p* = 0.05. Pearson Chi-square (χ^2^) test was used to compare significant difference of end-product toxicity on tomato seed germination and seedling growth in the fungus and control treatments, and the number of plants that were infested with the red spider mites in fungus and control composted materials. All computations were performed using PAST version 3.20 (Øyvind Hammer, Oslo, Norway) [33]. The values reported in this paper are mean ±SE (standard error). 

## 3. Results

### 3.1. Effect of Fungal Treatment on Compost Heap Temperature and Heap Humidity

Heap temperature showed significant differences between treatments at weeks 1, 7, 8, and 9 in the morning (DF; degree of freedom = 1, 10; *p* = 0.05), and weeks 7 and 9 in the afternoon (Table 1). 

### 3.2. Variation in Environmental Conditions (Relative Humidity and Ambient Temperature)

The data for environmental conditions were collected in the mornings and afternoons and are shown in Appendix A; Table A1. 

### 3.3. Heap Moisture

The fungal inoculum did not have any significant effect (*p* > 0.05) on heap moisture compared to the control at weeks 1, 3–4, 6–7, and 10–12 in both the morning and afternoon. However, there were significant differences (DF = 1, 10; *p* < 0.05) recorded in heap moisture in the morning at weeks 5 and 9, and afternoon at weeks 2 and 8 post-treatment (Table 2). 

### 3.4. pH Variations

There were no significant differences (*p* > 0.05) in pH of compost heaps with fungal-treated materials and the control at week one to 12 weeks for both the morning and afternoon readings, including the final end-product after composting (Table 3). An interesting similarity was observed in the end-product of composting, where the pH values were alkaline for both treatments, ranging from 7.5 to 7.6 (Table 4). 

### 3.5. Changes in the Color of the Compost during Composting

Variations in color from green raw materials in the initial stage of the experiment to brown (control) and dark brown (fungus-treated heaps) color at twelve weeks post treatment were observed between treatments. The Munsell Book of Colors [27] guideline classified the fungus-treated compost as 5 YR 3/3 (dark-brown color) and the control compost as 5 YR 4/4 (brown in color). 

### 3.6. Chemical Analysis of Compost: Macro-and Micro-Nutrients

Generally, there was no significant difference (*p* > 0.05) in the macro-and micro-nutrient levels in composted materials between treatments (Table 4).

### 3.7. Re-Isolation of the Fungus from the Compost End-Product 

**Figure 1:**
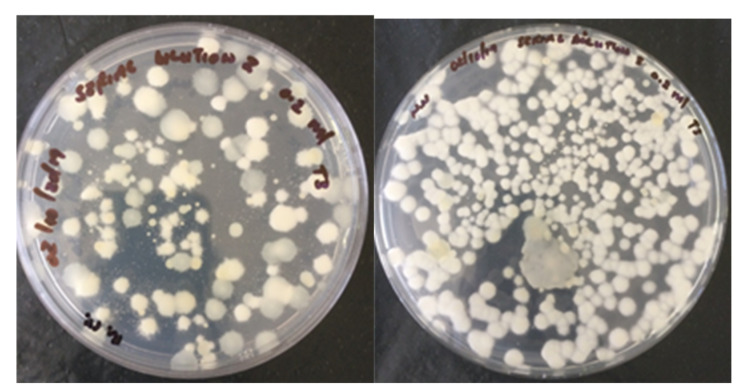
Re-isolation of *Clonostachys rosea* f. *catenula* isolate from the composted end-product materials obtained from fungus-treated heaps.

*Clonostachys rosea* f. *catenula* was successfully re-isolated from the composted materials that were exposed to fungal inoculum (Figure 1), but not from the control-treated heaps.

### 3.8. End-Product Toxicity—Germination and Seedling Test

When tested at a 25%:75% compost:sand ratio mix, composted materials from fungus-treated heaps were more favorable to the germination of tomato seeds, where a significant difference (*p* < 0.05) in seed germination (χ^2^ = 12.102; *p* = 0.0005) was obtained. We obtained 93% successful seed germination in the fungus inoculated compost compared with 68% in the control treatment. In the seedling test, no seedling was affected by the fungus or control treatment: 100% of seedlings were alive. Furthermore, no symptoms of yellowing and burning of leaf edges of the plants were observed on the tested seedlings (Table 5). 

### 3.9. Re-Isolation of Fungus from Tomato Tissues

**Figure 2:**
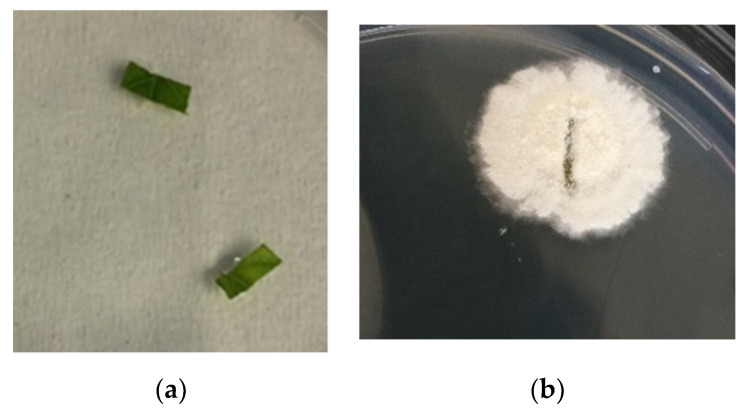
Re-isolation of *Clonostachys rosea* f. *catenula* isolated from leaf sections of tomato plants obtained from compost materials inoculated with *Clonostachys rosea* f. *catenula* and control treatment (no fungus) plated on PDA selective media. These samples were surface sterilized: (**a**) The petri dish corresponding to control samples, (**b**) and *C. rosea* f. *catenula* treated samples.

At 21 days after inoculation, *C. rosea* f. *catenula* was successfully re-isolated from 9 of 18 leaf sections placed onto PDA plates, representing 9 of 9 plants (100%) cultivated on composted end-product materials obtained from fungus-treated raw compost heaps (Figure 2). No fungal outgrowth of *C. rosea* f. *catenula* was observed in the plants grown in the control treatment compost and sand substrate. 

### 3.10. Protection of Tomato Plants against Red Spider Mite Infestation 

A lower *T. urticae* infestation level was observed among plants cultivated on composted materials from fungus treatment. However, overall, the *C. rosea* f. *catenula* isolate had a limited effect on *T. urticae* infestation occurrence on tomato plants grown in compost end-product substrate originating from control and fungus-treated heaps (χ^2^ = 0.96; *p* = 0.32). Of the 20 potted plants, cases of red spider mite infestations ranged between 6 and 9 for fungus and control treatments, respectively (Table 6). 

### 3.11. Effect of Fungus on Plant Tissue Nutrient Content

Generally, compost end-product from heaps inoculated with fungus had no significant effect (*p* > 0.05) on the tissue nutrient levels of plants compared with the control compost samples. However, K (32,750 mg/kg), Ca (19,250), and Na (2887.5 mg/kg) were higher in the leaf tissues of fungus-treated plants (DF = 1, 6; *p* < 0.05) compared to the control plants, which were 26,000 mg/kg K, 17425 mg/kg Ca, and 1950.5 mg/kg Na (Table 7). However, the Fe concentration in plants exposed to fungus-treated materials was in general lower compared to the control treatment compost (*p* > 0.05). 

## 4. Discussion

The inoculation of *C. rosea* f. *catenula* on composting heaps did not show varied effects on the physical parameter of heap temperature over time in this study. The highest weekly average temperature recorded in this study was 39.35 °C, and this was relatively low when compared to similar studies [34,35]; however, the heaps in the current study were small and in open air conditions. Gaur et al. [14] observed maximum temperatures of 45 °C and 51 °C during composting of a mixture of jowar stalk and wheat straw, and jamun leaves inoculated with *Aspergillus niger* and *Trichoderma viride*, respectively. However, the study by Sakar et al. [36] differed from the present study. For example, the study by Sakar et al. [36] did not show an increase in temperature in pits and earthen pots of compost wastes containing dung and market waste, and vegetable waste and dung, but when the ratio of 1:5 chopped rice straw was added to the waste, a heap temperature of 65.9 °C was observed after 24 h. The influence of the experimental conditions, such as heap size, ambient temperature and humidity, and turning frequencies on the composting process and the outcomes cannot be underestimated [32]. Possible reasons for the low temperature of composting in the present study could have been due to the high water content of the solid vegetable wastes and frequent turning of the heaps in open air [32]. Temperature is a key parameter in composting, as it indicates the state of decomposition and guarantees sanitation of the end-product [37]. The rise and drop in temperature suggests that the composting process might have gone through three phases: the mesophilic, thermophilic, and maturation phases. 

In this study, the data show that the effect of fungus on moisture content was not significant. However, the heap moisture content decreased in both treatments over time, from weeks 1 to 12. Moisture is considered as one of the important parameters affecting the composting process [15,38]. However, the moderate moisture content of 40–60% obtained in this study falls within the range of optimum moisture content for composting [39]. Ribeiro et al. [34] also expressed that values of 50 to 60% moisture content are ideal for efficient composting. Higher moisture content during composting causes waterlogging that might delay the composting process [36,40,41,42]. It is worth noting that the inoculation of composting materials with beneficial microorganisms has the potential to sustain the water content of heaps.

We also assessed the nutrient contents of the cabbage wastes in this study and found that N, P, K, Na, C, and B were not significantly influenced by exposing composting materials to a *C. rosea* f. *catenula* inoculum. These findings contradict those of Jusoh et al. [43], who reported higher levels of P and K content (*p* < 0.05) in a mixture of rice straw with a solution of effective microorganisms in comparison to those without microorganisms. This study recorded the same C/N ratio of 8:1 for both treatments in the end-product of composting (Table 4). Nevertheless, Kumar et al. [44] and Petric et al. [45] reported results with essential C/N values between 20 and 50. Previously, Mtimkulu [32] reported improved C/N ratio in treatments with spent wine filter material content (10:1; 13:1, and 10:1). A low C/N ratio suggests higher available nitrogen per carbon, and inert nitrogen [46]. Yang et al. [47], Wang et al. [48], and Rastogi et al. [49] observed decreased C/N ratios in the process of composting, which can be attributed to an elevated waste biodegradation (carbon) to mineralization (nitrogen) ratio.

Traditionally, C/N ratio is used to indicate the degree of maturity of compost [50,51]. Poincelot [52] suggested that a C/N ratio below 20 is indicative of mature compost. Therefore, based on our results, fungal-treated compost heaps and control-treated heaps were mature and complete at the end of the composting period. Similarly, the N and C in the plant tissue nutrients did not show a significant difference (*p* > 0.05) in both treatments; a higher C/N ratio (25:1) was obtained (Table 7). Fungus treated heaps appeared to have a dark brown color compared to the control heaps. This effect demonstrates that perhaps the fungal inoculum enhanced the decomposition of organic wastes. This affirmation correlates with the findings of Sakar et al. [36].

The existence of micronutrients, including some heavy metals, in the compost was determined at the end of the composting period, and interestingly, no effect on micro-nutrients was observed in the fungus-treated heaps compared with the control (Table 4). Overall, these results suggest that inoculation of cabbage wastes with the *C. rosea* f. *catenula* strain did not change the chemical characteristics of the final product of composting. Previously, a decrease in the status of nutrients had been obtained by Chaturvedi et al. [53], while, the findings of Jusoh et al. [43] indicated that the composting process of rice straw containing effective microorganisms tended to increase heavy metals (Cu and Zn). Previous research by Paré et al. [54] also explored the increased accumulation of heavy metals during the composting of biosolids. These trace metals are required in small quantities by plants, as they can be phytotoxic at high concentrations [52]. Paré et al. [54] confirmed that the extractability and exchangeability of some of these heavy metals could be reduced during the composting of biosolids and municipal solid wastes. 

No differences in the pH values of the treated heaps and the control heaps over the period of composting were recorded. The pH in this study ranged from 4.5–7.0. Previous studies showed that pH values ranging from 5.5 to 9.0 are suitable for the composting process, while values between 6.5 to 8.0 are thought to be most effective [55]. According to Chen et al. [41], a pH range from 6.8 to 7.3 is optimum for composting. Zhang and Sun [46] suggested that pH values from 5.5 to 8.0 are ideal for composting, whereas Bernal et al. [39] suggested that a pH value from 6.7 to 9.0 is optimum to promote good microbial activities. The pH of compost has a noticeable effect on the microbial populace, and it increases as a result of the decomposition of acids, which releases ammonium [15]. The pH values in the reports by Miller [29], Christian et al. [55], Sarkar et al. [36], and Ribeiro et al. [34] were closer to those observed in the present study (pH ranging between 7.0 and 7.6). This pH range of 7 to 7.6 is within the optimum range of 5.5 to 8.0 for compost pile materials with fungi [41]; thereby, suggesting that the compost in the current study was subjected to good oxidation. 

In this study, *C. rosea* f. *catenula* conidia were successfully re-isolated from the composted materials originating from fungus pre-treated heaps and from all tomato plants that were cultivated on the composted materials obtained from the fungus pre-treated heaps. These results suggest that the fungus was certainly persistent in the composted materials; thereby, enhancing endophytic quality of the composted materials. *C. rosea* f. *catenula* is potentially pathogenic to insects and antagonistic against phytopathogens. Hence, its presence should add value to organic composts. The re-isolation of *C. rosea* f. *catenula* from leaf sections of the cultivated tomato plants clearly demonstrated the *C. rosea* f. *catenula* strain used in this study was an efficient endophyte. The colonization of potted tomato plants by endophytic fungi has been reported previously [56]. Xia et al. [57] isolated six endophytic fungal isolates belonging to very diverse orders, such as Eurotiales, Pleosporales, Hypocreales, and Saccharomycetales. The recovery of *C. rosea* f. *catenula* from the leaves of the tomato plants, indicates the potential of these strains to become successful endophytic agents. Andrade-Linares et al. [58] also reported endophytic colonization by species belonging to the genera *Verticillium*, *Penicillium*, *Cladosporium*, *Fusarium,* and *Trichoderma*, which can also be isolated from stems. It is worth mentioning that successful colonization of fungal endophytes across different types of tissues is influenced by factors such as fungal species, fungal strain, and host [59,60,61], as well as plant species, growth stages, soil types, and environmental conditions [62]. Variability in the colonization efficiency of strawberry leaves [63] and potato tubers [64] with a different strain of *C. rosea* was previously reported. Nordström [65] reported variable colonization of tomato and *Arabidopsis thaliana* by a *C. rosea* f. *catenula* strain. Sutton et al. [20] also reported that *C. rosea* is a plant endophyte on different plant species and was isolated from roots and stems of soya beans (*Glycine max*), which were either inoculated with, or were grown in, soil infested with *C. rosea* [66]. Nordström [65] suggested that the rhizosphere can affect the establishment of *C. rosea.* In another study, it was reported that growth media has a greater influence on the level of plant endophytic colonization than the inoculation method [67].

*Clonostachys rosea* f. *catenula* was assessed against a tomato plant red spider mite infestation and the results showed that the fungus did not significantly reduce the insect infestation levels despite the high endophytism observed in this study, suggesting that many factors influence the efficacies of endophytic entomopathogens in vivo. Moloinyane and Nchu [68] did not observe a reduction of grapevine mealy bug infestations by the endophyte *Beauveria bassiana,* and they argued that factors such as host species, fungal strain, and insect species may influence the efficacy of endophytic entomopathogens on both the plant and the insect pest. However, inoculation method and the effect of endophytic colonization can be influenced by biotic and abiotic factors, such as growth substrate, plant species and age, fungal species and inoculum density [59,66,69]. For instance, Tefera and Vidal [59] demonstrated that despite the inoculation method used, *B. bassiana* colonized the entire parts of sorghum plants. However, a study by Posada et al. [69] showed that plants species, fungal isolate, and the inoculation methods used influenced the endophytic colonization by *B. bassiana*. Some studies have demonstrated fungal endophytes reduces insect infestations [16,70,71,72,73,74]. For instance, the effect of environmental changes on colonization of corn by the fungal isolate *B. bassiana* was reported by Bing and Lewis [75] and its effect on targeted pests (European corn borer larvae) *Ostrinia nubilalis* (Hübner). However, others did not find any benefit of endophytic fungal colonization on insect infestations [68]. It appears many other factors moderated the fungus–plant–herbivore relationship, which research has shown is quite complex. It is worth noting that this fungus is known as an antagonist, and which is widely used as a biocontrol agent for divergent plant pathogens, including plant pathogenic fungi such as *Alternaria* and *Fusarium* species [76].

Results of this study showed a superior germination of tomato seeds sown in composted materials from heaps that were inoculated with *C. rosea* f. *catenula* during composting, and no phytotoxicity was observed on percentage seedling growth in both treatments. The increased germination in treated compost, as well as the increased seedling growth, may have been due to the presence of the fungus. A recent study showed that endophytic fungi can produce phytohormones, particularly gibberellins, that alleviate the harmful effects of abiotic stresses and enhance crop growth [77]. According to Davies [78], these phytohormones can regulate various developmental and physiological processes in plants such as seed germination, seedling development, stem and leaf growth, and flower and fruit growth. Previous studies reported on plant-growth promoting fungi (PGPF) from various genera including, *Thrichoderma*, *Fusarium,* and *Penicillium*, which can be beneficial in several crop plants [79,80]. A similar study has been reported, wherein *Penicillium oxalicum* (*Pennisetum glaucum* (L.) R. Br.) enhanced the seed germination and seedling vigor of Pearl millet [81]. However, the authors indicated that the efficacy of the endophytic fungus may vary significantly among treatments. Similarly, [79] observed enhanced seed germination and seedling vigor where seed priming with a conidial suspension of PGPF *T*. *harzianum* was used compared to a non-primed control in sunflower (*Helianthus annuus* L.). Moreover, Jogaiah et al. [82] demonstrated enhanced seed germination and seedling vigor in seeds of tomato treated with a conidial suspension of different PGPF. Murali et al. [83] also demonstrated the use of these microbes as inoculants to treat seeds for germination and seedling growth. Herrera et al. [84] found that seeds sown on a media composition of white peat mixed with municipal solid waste compost had better quality seedlings than those grown with standard peat mixtures. The compost:river sand ratio (25% treated compost and 75% course river sand) was a suitable treatment for the vegetative growth of seedlings in both treatments (Table 5). 

This study suggests that inoculating organic waste materials with endophytic entomopathogenic *C. rosea* f. *catenula* conidia could enhance the rate of decomposition of organic waste heaps, help to produce high quality composted materials rich in endophytes, and that composting materials could serve as a substrate for storing entomopathogenic fungi for long periods, and for fungal application in the field. The finished end-product from the fungus-inoculated heaps could improve the physical stability, chemical constituent richness, and promote plant vigor and growth enhancement. This compost end-product contains plant-growth promoting and protection properties and could substitute chemical fertilizers and synthetic pesticides, inhibit toxic chemicals to crop plants, and be recommendable in agricultural application. Future evaluation of enzymes could help to better understand the mechanism through which the fungus influences composting, changes in microbial species and populations over time, and identify the specific microbes involved. Assessment of phytotoxic chemicals of these fungi in composted materials could help understand the potential risk they may have in the cultivation of plants. 

## 5. Conclusions

In conclusion, this is the first report of the direct influence of *C. rosea* f. *catenula* on the composting of solid organic vegetable wastes and the tissue colonization of tomato plants. *Clonostachys rosea* f. *catenula* had no effect on the chemical composition of the end-product of composting of solid vegetable waste compared to the control treatment over the period of composting. However, the *C. rosea* f. *catenula* treatment enhanced the germination of tomato seeds and improved the physical characteristics of the composts. The *C. rosea* f. *catenula* strain did not confer protection against *T. urticae* infestation of tomatoes. Interestingly, the conidia of *C. rosea* f. *catenula* persisted in the composted materials for several months and was able to endophytically colonize tomato seedlings. We propose that organic wastes should be used as substrates for long term storage of endophytic entomopathogenic fungi. These findings also contributed to our understanding of the mechanisms involved in the fungus–plant residue–insect relationship. The effects of fungal inoculants on changes in enzyme activities during composting should be considered in the future. 

## Figures and Tables

**Table 1:** Variation in heap temperature (mean ± SE) following exposure to *Clonostachys rosea* f. *catenula* inoculum and control treatment (no fungus) during the composting of raw vegetable waste over a 12-week period of composting.

Fungal Treatment			Control Treatment	
Weeks	Morning	Afternoon	Morning	Afternoon
1	35.12 ± 0.6 b	36.58 ± 0.9 a	39.35 ± 0.2 a	36.28 ± 0.3 a
2	26.92 ± 1.5 a	25.88 ± 0.2 a	28.68 ± 0.2 a	26.83 ± 0.3 a
3	23.77 ± 0.5 a	25.62 ± 0.5 a	24.08 ± 0.1 a	29.53 ± 2.0 a
4	28.98 ± 1.7 a	30.53 ± 0.6 a	27.9 ± 0.2 a	31.98 ± 0.6 a
5	29.12 ± 1.6 a	31.62 ± 0.7 a	29.63 ± 1.5 a	33.2 ± 0.4 a
6	28.53 ± 0.4 a	34.48 ± 0.9 a	28.72 ± 0.3 a	34.17 ± 0.3 a
7	24.37 ± 0.3 b	27.76 ± 0.4 b	26.8 ± 0.3 a	31.22 ± 0.8 a
8	26.9 ± 0.8 b	29.62 ± 2.3 a	29.15 ± 0.5 a	29.55 ± 0.8 a
9	27.37 ± 0.3 b	31.05 ± 0.4 b	29.45 ± 0.2 a	34.23 ± 0.8 a
10	28.82 ± 1.0 a	32.58 ± 0.9 a	30.68 ± 1.0 a	33.00 ± 0.8 a
11	27.23 ± 0.9 a	31.62 ± 0.9 a	28.65 ± 0.6 a	34.33 ± 0.9 a
12	29.03 ± 2.0 a	33.22 ± 0.9 a	33.37 ± 1.0 a	34.55 ± 0.9 a

Values are means ± SE (standard error). Values followed by the same lowercase letters in the same row do not show significant difference at *p* > 0.05 following comparison using the Tukey test. Means with the same letters are not significantly different.

**Table 2:** Changes in heap moisture reading (mean ± SE) following exposure to *Clonostachys rosea* f. *catenula* inoculum and control treatment (no fungus) during composting of raw cabbage waste.

Fungal Treatment			Control Treatment	
Weeks	Morning	Afternoon	Morning	Afternoon
1	52.97 ± 22.2 a	46.65 ± 1.8 a	51.92 ± 1.7 a	40.98 ± 7.5 a
2	43.53 ± 3.3 a	48.5 ± 2.6 a	39.92 ± 1.9 a	40.96 ± 1.5 b
3	44.23 ± 4.9 a	46.97 ± 3.9 a	39.33 ± 2.7 a	40.3 ± 3.9 a
4	44.87 ± 2.9 a	40.58 ± 4.9 a	42.23 ± 3.0 a	47.23 ± 3.4 a
5	43.2 ± 0.8 a	49.23 ± 2.6 a	52.1 ± 3.7 b	52.23 ± 1.2 a
6	44.45 ± 1.7 a	43.42 ± 1.5 a	40.1 ± 15.5 a	45.45 ± 1.0 a
7	37.9 ± 1.2 a	38.42 ± 3.1 a	42.52 ± 2.2 a	37.85 ± 1.6 a
8	44.72 ± 1.1 a	49.47 ± 1.2 a	38.87 ± 3.9 a	44.45 ± 2.1 b
9	22.15 ± 1.6 a	19.48 ± 0.9 a	17.9 ± 0.7 b	21.07 ± 1.3 a
10	0.00 ± 0.0 a	0.00 ± 0.0 a	0.00 ± 0.0 a	0.00 ± 0.0 a
11	12.07 ± 1.4 a	0.00 ±0.0 a	11.37 ± 0.9 a	0.00 ± 0.0 a
12	0.00 ± 0.0 a	0.00 ± 0.0 a	0.00 ± 0.0 a	0.00 ± 0.0 a

Values are means% ± SE (standard error). Values followed by the same lowercase letters in the same row do not show a significant difference (*p* > 0.05) following a comparison using the Tukey test. Means with the same letters are not significantly different.

**Table 3:** pH variation (mean ± SE) following exposure to *Clonostachys rosea* f. *catenula* inoculum and control treatment (no fungus) during the composting of raw cabbage waste over a 12-week period of composting.

Fungal Treatment			Control Treatment	
Weeks	Morning	Afternoon	Morning	Afternoon
1	4.50 ± 0.1 a	4.70 ± 0.1 a	4.60 ± 0.1 a	4.80 ± 0.1 a
2	5.30 ± 0.2 a	5.00 ± 0.2 a	5.20 ± 0.0 a	5.60 ± 0.4 a
3	5.20 ± 0.2 a	4.90 ± 0.2 a	5.40 ± 0.2 a	5.30 ± 0.1 a
4	5.00 ± 0.1 a	5.20 ± 0.3 a	5.00 ± 0.1 a	4.80 ± 0.2 a
5	5.10 ± 0.0 a	4.60 ± 0.1 a	4.50 ± 0.2 a	4.50 ± 0.1 a
6	5.40 ± 0.3 a	5.00 ± 0.1 a	5.20 ± 0.1 a	4.90 ± 0.0 a
7	5.30 ± 0.1 a	5.3 ± 0.2 a	5.00 ± 0.5 b	5.30 ± 0.1 a
8	5.00 ± 0.1 a	4.7 ± 0.1 a	5.30 ± 0.3 a	5.00 ± 0.1 a
9	6.10 ± 0.1 a	6.2 ± 0.0 a	6.30 ± 0.0 a	6.10 ± 0.1 a
10	7.00 ± 0.0 a	7.00 ± 0.0 a	7.00 ± 0.0 a	7.00 ± 0.0 a
11	6.40 ± 0.1 a	7.00 ± 0.1 a	6.50 ± 0.0 a	7.00 ± 0.0 a
12	7.00 ± 0.0 a	7.00 ± 0.0 a	7.00 ± 0.0 a	7.00 ± 0.0 a

Values are means ± SE (standard error). Values followed by the same lowercase letters in the same row do not show a significant difference at (*p* > 0.05) following comparison by using the Tukey test. Means with the same letters are not significantly different.

**Table 4:** The effect of treatment with *Clonostachys rosea* f. *catenula* on the chemical composition of raw cabbage compost over the 12-week period of composting.

Parameters	Fungus Treatment	Control Treatment
pH	7.60 ± 0.0 a	7.50 ± 0.0 a
N mg/kg	22,540.00 ± 1111.1 a	24,660.00 ± 2182.1 a
P mg/kg	6400.00 ± 158.1 a	5860.00 ± 254.2 a
K mg/kg	6740.00 ± 958.4 a	7400.00 ± 931.1 a
Ca mg/kg	39,880.00 ± 446.5 a	38,220.00 ± 950.5 a
Mg mg/kg	2960.00 ± 67.8 a	3180.00 ± 345.5 a
Na mg/kg	2099.60 ± 571 a	1680.90 ± 217.6 a
Mn mg/kg	34.90 ± 1.5 a	36.20 ± 0.9 a
Fe mg/kg	199.92 ± 14.7 a	210.30 ± 6.6 a
Cu mg/kg	6.90 ± 1.1 a	8.32 ± 1.3 a
Zn mg/kg	75.04 ± 6.8 a	68.54 ± 8.6 a
B mg/kg	26.83 ± 1.9 a	27.90 ± 2.7 a
C mg/kg	221.34 ± 23.3 a	182.56 ± 12.8 a
C/N ratio	8:1 ± 0.4 a	8:1 ± 0.2 a

Values are means ± SE. (standard error). Values followed by the same lowercase letters in the same row do not show a significant difference at *p* > 0.05) following a comparison by using the Tukey test. Means with same the letters are not significantly different.

**Table 5:** Seed germination and seedling test of *Solanum lycopersicum* plants cultivated in growth medium made up of the end-products of composting that were obtained from *Clonostachys rosea* f. *catenula*-containing compost heaps and control compost heaps (no fungus). The growth medium was composed of 25% composted materials and 75% sand.

Percentage Seed Germination		Percentage Seedling Growth	
Treatment	Control	Treatment	Control
93 a	68 b	100 a	100 a

Means with the same lowercase letters in the same row in either seed germination or seedling growth are not significantly different following Pearson Chi-square test (χ^2^) at a *p* = 0.05 level of significance for seed germination and seedling growth. Means with the same letters are not significantly different.

**Table 6:** Infestation occurrence of *Tetranychus urticae* on tomato seedlings cultivated in substrate mix containing composted materials from either organic waste heaps that were inoculated with *Clonostachys rosea* f. *catenula* or control treated heaps.

	Plants Cultivated on Fungus-Treated Heaps	Plants Cultivated on Control-Treated Heaps
Number of plants infested by *T. urticae*	6/20	9/20

Samples of plants that were infested with red spider mites from the total number of planted tomato seedlings.

**Table 7:** Tissue nutrient contents (mean ± SE) of leaves of *Solanum lycopersicum* plants grown for eight weeks under greenhouse conditions and that were exposed to compost originating from control compost heaps and *Clonostachys rosea* f. *catenula* inocula exposed compost heaps.

Parameters	Treatment	Control
*Macronutrients*		
N mg/kg	16,575.00 ± 1527.2 a	17,600.00 ± 1987.0 a
P mg/kg	1500.00 ± 91.3 a	2200.00 ± 385.1 a
K mg/kg	32,750.00 ± 3637.2 a	26,000.00 ± 4830.5 a
Ca mg/kg	19,250.00 ± 1600.8 a	17,425.00 ± 2917.9 a
Mg mg/kg	5000.00 ± 430.1 a	5650.00 ± 1278.3 a
Na mg/kg	2887.50 ± 617.2 a	1950.50 ± 355 a
*Micronutrients*		
Mn mg/kg	59.10 ± 9.1 a	49.70 ± 11.9 a
Fe mg/kg	120.50 ± 14.9 a	153.85 ± 65.4 a
Cu mg/kg	4.20 ± 0.5 a	4.35 ± 0.9 a
Zn mg/kg	50.10 ± 1.5 a	53.80 ± 10.6 a
B mg/kg	56.37 ± 6.9 a	46.58 ± 4.8 a
C mg/kg	413.30 ± 1.3 a	414.10 ± 6.5 a
C/N ratio	25:1 ± 2.6 a	24:1 ± 2.6 a

Values are means ± SE (standard error). Values followed by the same letter in the same row do not show a significant difference at *p* > 0.05 following a comparison by using the Tukey test. Means with the same letters are not significantly different.

## Appendix A

**Table A1:** Changes in relative humidity (mean ± SE %) and ambient temperature readings (mean ± SE °C) during the composting of raw vegetable wastes over a 12-week period of composting.

Relative Humidity			Ambient Temperature	
Weeks	Morning	Afternoon	Morning	Afternoon
1	50.2 ± 3.5	52.84 ± 5.1	30.52 ± 3.6	31.16 ± 4.7
2	54.42 ± 1	57.1 ± 1.7	25.04 ± 1.0	24.18 ± 1.9
3	55.54 ± 4.9	53.96 ± 2.7	24.66 ± 0.9	23.12 ± 0.4
4	60.76 ± 2.7	56.16 ± 3.0	26.26 ± 1	26.32 ± 1.3
5	59.4 ± 3.1	56.32 ± 5.7	25.68 ± 0.7	24.78 ± 0.6
6	63.3 ± 2.6	66.04 ± 3.4	28.08 ± 1.4	26.34 ±1.2
7	53.36 ± 1.5	51.6 ± 2.4	28.1 ± 0.6	29.42 ±1.4
8	55.36 ± 2.0	53.74 ± 2.7	27.68 ± 0.6	26.62 ± 0.8
9	59.18 ± 3.6	60.02 ± 2.4	28.08 ± 1.6	27.68 ± 0.3
10	59.64 ± 0.3	57.06 ± 2	26.34 ± 0.6	27.76 ± 0.5
11	60.76 ± 2.7	56.06 ± 1.8	27.2 ± 1.0	28.26 ± 0.6
12	51.5 ± 1.4	47.74 ± 2.6	28.66 ± 0.6	27.18 ± 0.6
